# Effect of Separate and Combined Toxicity of Bisphenol A and Zinc on the Soil Microbiome

**DOI:** 10.3390/ijms23115937

**Published:** 2022-05-25

**Authors:** Magdalena Zaborowska, Jadwiga Wyszkowska, Agata Borowik, Jan Kucharski

**Affiliations:** Department of Soil Science and Microbiology, University of Warmia and Mazury in Olsztyn, Plac Łódzki 3, 10-727 Olsztyn, Poland; m.zaborowska@uwm.edu.pl (M.Z.); agata.borowik@uwm.edu.pl (A.B.); jan.kucharski@uwm.edu.pl (J.K.)

**Keywords:** bisphenol A, zinc, soil enzymes, soil microbiome, biodiversity, humic acid

## Abstract

The research objective was established by taking into account common sources of soil contamination with bisphenol A (B) and zinc (Zn^2+^), as well as the scarcity of data on the effect of metabolic pathways involved in the degradation of organic compounds on the complexation of zinc in soil. Therefore, the aim of this study was to determine the spectrum of soil homeostasis disorders arising under the pressure of both the separate and combined toxicity of bisphenol A and Zn^2+^. With a broad pool of indicators, such as indices of the effect of xenobiotics (IF_X_), humic acid (IF_H_), plants (IF_P_), colony development (CD), ecophysiological diversity (EP), the Shannon–Weaver and the Simpson indices, as well as the index of soil biological fertility (BA_21_), the extent of disturbances was verified on the basis of enzymatic activity, microbiological activity, and structural diversity of the soil microbiome. A holistic character of the study was achieved, having determined the indicators of tolerance (IT) of *Sorghum Moench* (S) and *Panicum virgatum* (P), the ratio of the mass of their aerial parts to roots (PR), and the SPAD leaf greenness index. Bisphenol A not only failed to perform a complexing role towards Zn^2+^, but in combination with this heavy metal, had a particularly negative effect on the soil microbiome and enzymatic activity. The NGS analysis distinguished certain unique genera of bacteria in all objects, representing the phyla *Actinobacteriota* and *Proteobacteria*, as well as fungi classified as members of the phyla *Ascomycota* and *Basidiomycota*. *Sorghum Moench* (S) proved to be more sensitive to the xenobiotics than *Panicum virgatum* (P).

## 1. Introduction

The widespread industrial use of bisphenol A (B) started in the United States of America in 1957, and involved the production of polycarbonates and epoxy resins. Currently, this chemical can be found in protective coatings of food containers, as well as in paints, glues, and electronic laminates [1,2]. The Environmental Protection Agency had a good reason to classify bisphenol A as a High Production Volume (HPV) chemical [3]. It is estimated that in 2022, the production of bisphenol A in the European Economic Area will amount to approximately 10.6 × 10^9^ kg. It is forecast, however, that the global production of bisphenol A in 2023 will reach 7.3 × 10^9^ kg [4], with China being one of the major producers of this chemical [5]. Such an immense interest in this phenolic compound as a monomer arises from the fact that bisphenol A in polymer matrices delays the oxidative degradation of plastic materials exposed to ultraviolet radiation [6], in addition to acting as a stabilizer and antioxidant in PVC, as well as a precursor in the production of a brominated flame retardant, known as tetrabromobisphenol A (TBBPA) [7]. Although bisphenol A is rapidly biotransformed and excreted in the urine as BPA-G [8], exposure to this chemical is linked to infertility, diabetes, or abnormalities in brain development, including autism, the latter being attributed to bisphenol A interfering with synaptogenesis and neurogenesis [9,10]. Bisphenol A strongly destabilizes the functioning of the endocrine system (EDC), thereby contributing to a significant economic burden, estimated at EUR 163 billion in the EU and at EUR 326 billion in the USA [11,12]. Nevertheless, the gravest problem is the high incidence and huge amount of bisphenol A in environmental media, including sewage sludge, where its estimated quantity in Europe reaches 95,000 µg kg^−1^ d.w. of sludge [13]. Sewage sludge is also the main source of pollution of agricultural land with heavy metals, such as zinc [14]. It is estimated that 20 million hectares (ha) of farmland globally are contaminated with heavy metals, which has become an alarming problem, especially in developing countries. It is claimed that the volume of sewage sludge rose from 11.5 million Mg in 2010 to 13 million Mg in 2020 [15]. The increase in soil pollution with zinc is correlated with mass industrialization and urbanization, which stimulate the demand for heavy metals in automotive fuels, explosive materials, batteries, aeronautics, or in the steel industry [16,17,18]. Significantly, apart from being a large bisphenol A manufacturer, China ranks first as the world’s zinc producer, with an output of around 5 × 10^9^ kg Zn annually [19]. In the human body, zinc is known to participate in the synthesis of over 70 enzymes essential for the growth of cerebral cells [20]. This notwithstanding, zinc excess leads to neurodegenerative disorders, such as Alzheimer’s disease, depression, Parkinson’s disease, Huntington’s disease, or prion disease [21].

The interference of zinc and bisphenol A with the soil microbiome is a consequence of the dispersion of these substances in the environment. Heavy metals, including zinc, do not degrade in soil and therefore, persistently exert selective pressure on microorganisms [22]. The tolerance of microorganisms to heavy metals is tightly linked with the presence of a wide array of genes such as: *ars*B, *zra*R, *hyd*H, *znu*C/*yeb*M, *tro*B [23], or *aad*A, *str*B, *bla*CMY, and *ttg*B, *acr*F [24]. Integrons and transposons, which play a key role in the formation of microbial resistance through horizontal gene transfer (HGT) [25], are also significant. Zinc not only decreases the abundance and diversity of the soil microbiome by disrupting cellular metabolism, but also eventually inhibits the activity of soil enzymes [26,27]. An important role in the degradation of bisphenol A in soil is played by genes located in microbial plasmids or chromosomes encoding the enzymes involved in biodegradation pathways. These include *bam*A, pheA2A1, *Phe*R, and *ncg*l2587 [28,29,30]. Nonetheless, bisphenol A has an adverse effect on both the genotypic and phenotypic diversity of the soil microbiome and on the activity of soil enzymes [31].

The range of toxicological effects of the mentioned xenobiotics include the interactions of bisphenol A and zinc with plants. The toxic effects arising from the bioaccumulation of bisphenol A are particularly evident in plant roots [32]. The content of zinc in dry matter above 300 mg kg^−1^ is considered to be toxic [33]. The phytotoxic effect of zinc may cause structural and developmental disturbances in plant cells [34].

Remediation of soils contaminated with heavy metals, unlike those polluted with organic substances, consists mostly of the immobilization of these pollutants or their conversion to less toxic compounds [26]. The remediation of soils polluted with zinc often employs passivation methods, of which the most promising one consists of the application of humic acid (HA) [35,36]. Humic acid with complexation sites (carboxylic and hydroxyl functional groups) is effective in reducing the bioavailability and mobility of zinc in soil and its absorption by plants [37].

A broad perusal of the research reports concerning sorption and the consequences of soil pollution with heavy metals and bisphenol A dispersed in the environment revealed the lack of data on the reaction of organic contaminants to the soil polluted with zinc, and vice versa [38]. For this reason, extensive research has been launched to determine the response of the soil microbiome, including the activity of soil enzymes, as well as the counts and diversity of microorganisms, to the separate and combined toxicity of bisphenol A and zinc. Preventative measures have also been taken to eliminate the potential inhibitory effect of bisphenol A and zinc by applying humic acid. A holistic character of the research was achieved owing to the determination of the impact of these two xenobiotics on the growth and development of sorghum (*Sorghum Moench*) and switchgrass (*Panicum virgatum*).

## 2. Results

### 2.1. Enzyme Activity

**Figure 1:**
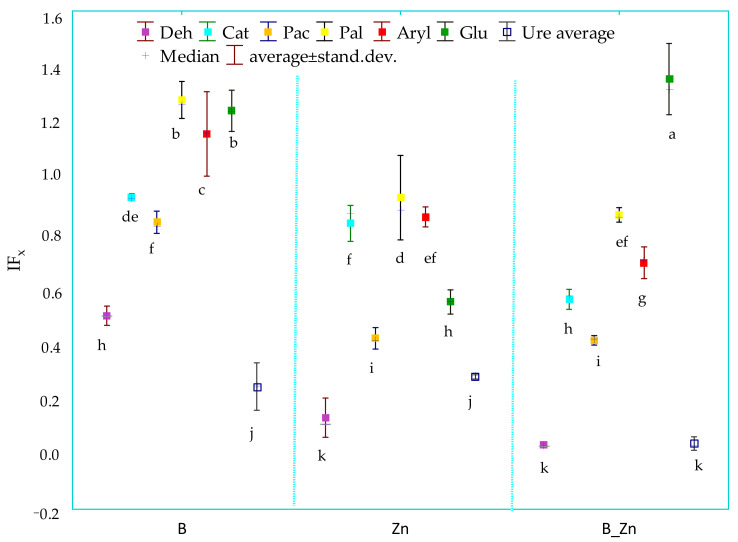
The coefficient of the influence of xenobiotics (IF_X_) on the activity of dehydrogenases (Deh), catalase (Cat), urease (Ure), acid phosphatase (Pac), alkaline phosphatase (Pal), arylsulfatase (Aryl), and β-glucosidase (Glu) in unsown soil contaminated with B, and Zn^2+^, B + Zn^2+^; B—bisphenol A; Zn—zinc ion (Zn^2+^). Homogeneous groups denoted with letters (a–k) were calculated for all xenobiotics.

The research results showed that the pollutants applied to soil, both bisphenol A and Zn^2+^, as well as their compilation, had adverse effects on the biochemical activity of the soil, although the sensitivity of individual enzymes varied (Table S1). The soil equilibrium was significantly disturbed by the pressure of both Zn^2+^ alone and combined with bisphenol A. The soil contaminated with B + Zn^2+^ most severely inhibited the activity of Deh (97%), Ure (96%), and Pac (60%), compared with controls. Zn^2+^ as a pollutant had a much greater inhibitory power than bisphenol A. The application of this heavy metal reduced the activity of Deh and Pac by 90% and 58%, respectively, whereas bisphenol A decreased the activity of these enzymes by 50% and 16%, respectively (Table S1). Contrary to expectations, some stimulating effect of bisphenol A on the activity of Pal and Glu was observed, as revealed by the influencing factor of the xenobiotics (IFx) with respect to these two enzymes (Figure 1).

**Figure 2:**
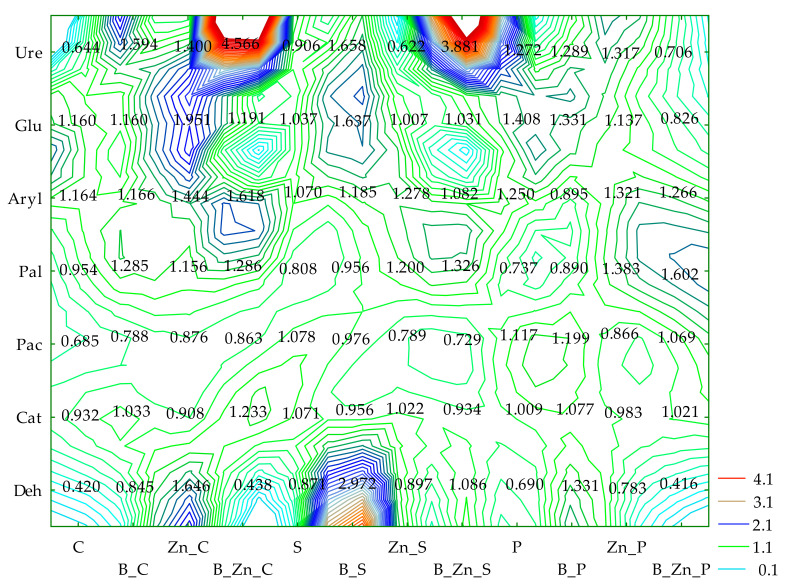
The coefficient of the influence of humic acid (IF_H_) on the activity of dehydrogenases (Deh), catalase (Cat), urease (Ure), acid phosphatase (Pac), alkaline phosphatase (Pal), arylsulfatase (Aryl), and β-glucosidase (Glu) in soil uncontaminated and contaminated with B, Zn^2+^, and B + Zn^2+^; S—*Sorghum Moench*; P—*Panicum virgatum*; C—uncontaminated soil; B—bisphenol A; Zn—ion zinc (Zn^2+^).

Based on the IFx values obtained, and in terms of the sensitivity of enzymes to the tested pollutants and their combinations, they can be ordered as follows: bisphenol A (B): Ure > Deh > Pac > Cat > Aryl > Glu > Pal; Zn^2+^: Deh > Ure > Pac > Glu > Cat > Aryl > Pal; B + Zn^2+^: Deh > Ure > Pac > Cat > Aryl > Pal > Glu. Humic acid applied in this experiment as a potentially biostimulating substance had a notably beneficial effect on Ure in unsown objects (IF_H_ = 4.566) exposed to the combined contamination of B and Zn^2+^ and in the parallel objects sown with sorghum (S) (IF_H_ = 3.881) (Figure 2).

**Figure 3:**
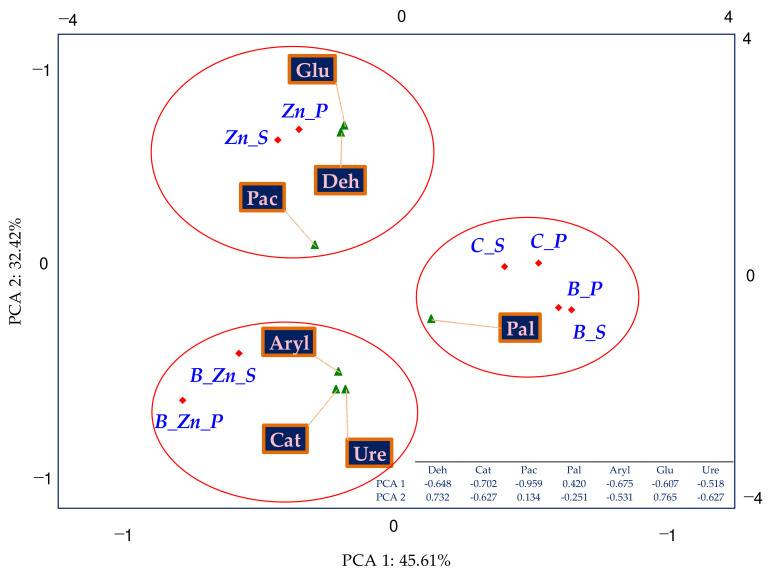
The coefficient of the influence of plants (IF_P_) on the activity of dehydrogenases (Deh), catalase (Cat), urease (Ure), acid phosphatase (Pac), alkaline phosphatase (Pal), arylsulfatase (Aryl), and β-glucosidase (Glu) in uncontaminated and contaminated soil B, Zn^2+^, and B + Zn^2+^—PCA method; S—*Sorghum Moench* (S) and P—*Panicum virgatum*, C—uncontaminated soil, B—bisphenol A, Zn—ion zinc (Zn^2+^).

**Figure 4:**
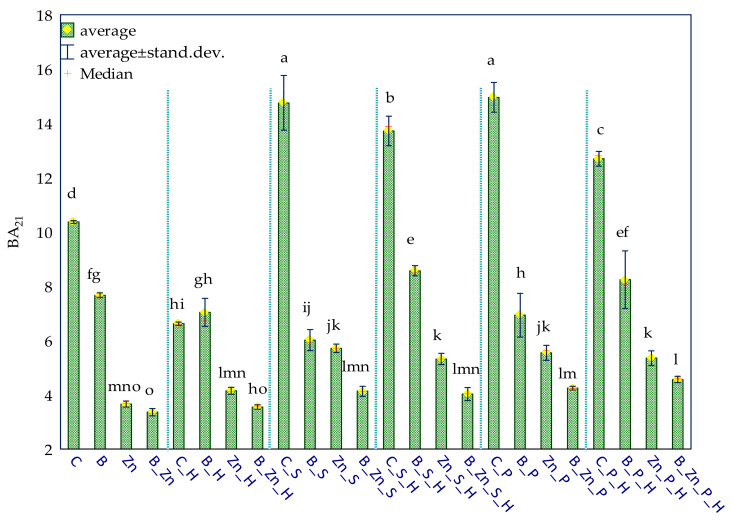
Biochemical fertility index (BA_21_) of soil uncontaminated and contaminated with B, Zn^2+^, and B + Zn^2+^; S—*Sorghum Moench* (S) and P—*Panicum virgatum*; C—uncontaminated soil; B—bisphenol A; Zn—ion zinc (Zn^2+^). Homogeneous groups denoted with letters (a–o) were calculated for all BA_21_ index values.

The effect of sowing the soil with plants (IF_P_), both sorghum (S) and switchgrass (P), on the biochemical activity of soil was associated with the contamination variants (Figure 3). The resultant tendencies were displayed through PCA multidimensional analysis. Based on the distribution of all cases on the PCA map, it can be concluded that the cropping of soil contaminated by Zn^2+^ with sorghum (S) or switchgrass (P) had a positive effect on the activity of Deh and Glu; when soil was polluted with B + Zn^2+^, this positive effect was manifested on the activity of Cat, Aryl, and Ure, and in the soil exposed to the pressure of bisphenol A, the cultivation of the plants positively affected the activity of Pal. The analysis of the soil fertility biochemical index (BA_21_) demonstrated the negative effect of the pollution treatments on this parameter, with bisphenol A having the mildest inhibitory influence (Figure 4). The biostimulation of unsown soil with humic acid did not bring about the expected outcomes. However, it contributed to the improved fertility of the soil cropped with sorghum (S) or with switchgrass (P) exposed to 1000 mg B kg^−1^ d.m. of soil.

### 2.2. Response of Microorganisms to Soil Contamination with Bisphenol A and Zinc

#### 2.2.1. Culturable Microorganisms

**Figure 5:**
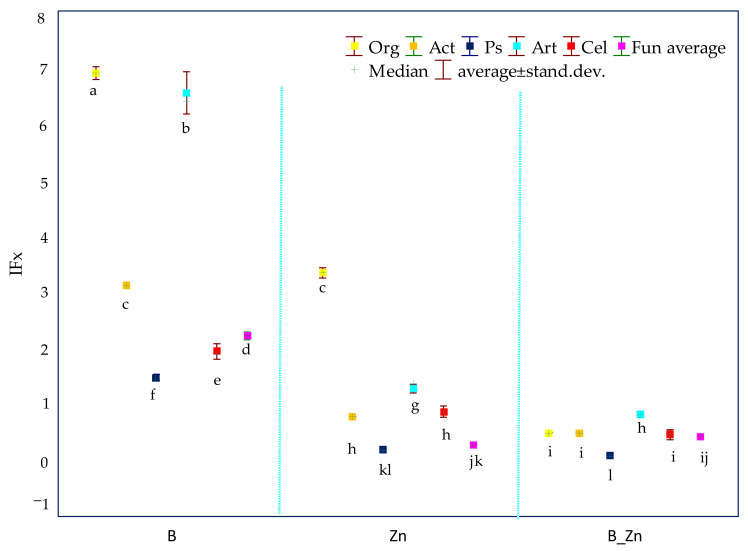
Reaction of soil microorganisms to unsown soil contaminated with B, Zn^2+^ and B + Zn^2+^ on the basis of the xenobiotic influence coefficient (IF_X_); organotrophic bacteria (Org), actinomycetes (Act), fungi (Fun), *Pseudomonas* sp. (Ps), *Arthrobacter* sp. (Art), cellulolytic bacteria (Cel); B—bisphenol A; Zn—zinc ion (Zn^2+^). Homogeneous groups denoted with letters (a–l) were calculated for all xenobiotics.

Based on the analysis of the research results, it can be concluded that both bisphenol A, Zn^2+^ and B + Zn^2+^ significantly moderated the microbiological activity of soil (Table S2). Of note is the fact that 1000 mg B kg^−1^ d.m. of soil stimulated the multiplication of all analyzed groups of microorganisms. The exposure to soil contaminated with bisphenol A caused an over 7-fold increase in the count of organotrophic bacteria, a 6-fold increase in bacteria of the genus *Arthrobacter*, a 3–fold increase of actinomycetes, and a 2-fold increase in fungi and cellulolytic bacteria, while the count of *Pseudomonas* sp. increased by 47% in relation to the controls. The strongest inhibitory effect on the counts of the seven groups of microorganisms was produced by the soil application of B + Zn^2+^. The microorganisms which were most sensitive to the compilation of these xenobiotics were *Pseudomonas* sp., fungi, and cellulolytic bacteria. Their counts under the pressure of the combined toxicity of bisphenol A and Zn^2+^ decreased by 91% for Pseudomonas sp., by 58% for fungi, and by 54% for cellulolytic bacteria. The exposure to 1000 mg Zn^2+^ kg d.m. of soil caused a significant decrease in the counts of *Pseudomonas* sp. (81%), fungi (72%), and actinomycetes (23%), while the count of bacteria of the genus *Arthrobacter* increased by 28%, in comparison with the controls (Table S2). Based on the values of the IF_X_, and considering the sensitivity of the groups of microorganisms to the pollution variants, the microorganisms can be ordered as follows: bisphenol A: Ps > Cel > Fun > Act > Art > Org; Zn^2+^: Ps > Fun > Act > Cel > Art > Org; B + Zn^2+^: Ps > Fun > Cel > Act = Org > Art (Figure 5).

**Figure 6:**
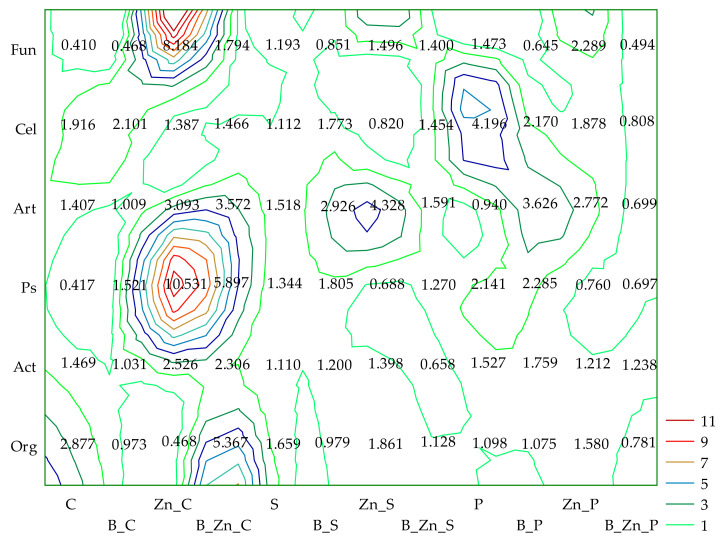
The coefficient of the influence of humic acid (IF_H_) on the number of organotrophic bacteria (Org), actinomycetes (Act), fungi (Fun), *Pseudomonas* sp. (Ps), *Arthrobacter* sp. (Art), and cellulolytic bacteria (Cel) in soil uncontaminated and contaminated with B, Zn^2+^, and B + Zn^2+^; S—*Sorghum Moench*, P—*Panicum virgatum*; C—uncontaminated soil; B—bisphenol A; Zn—ion zinc (Zn^2+^).

Having determined the humic acid influence index (IF_H_) values in relations to the analyzed parameter, it was possible to detect the spectacular mollifying effect of humic acid on the counts of *Pseudomonas* sp. (IF_H_ = 5.897) and organotrophic bacteria (IF_H_ = 5.367) in soil polluted with B + Zn^2+^ and on bacteria of the genus *Pseudomonas* in soil submitted to pressure Zn^2+^ (IF_H_ = 10.531) (Figure 6).

**Figure 7:**
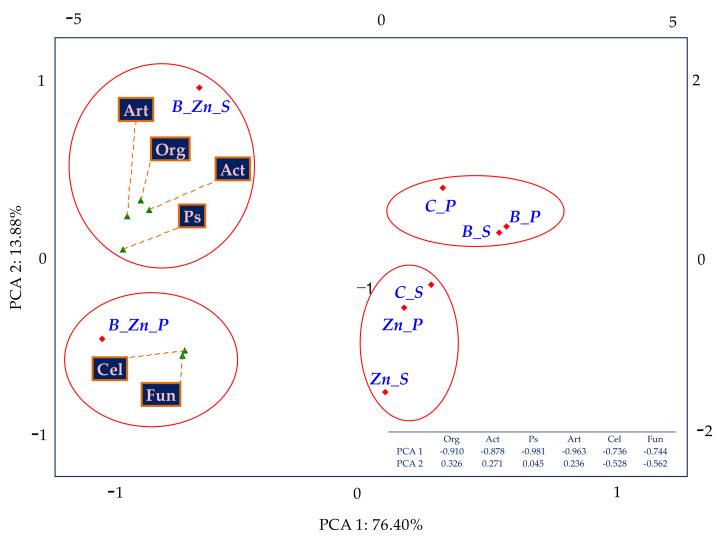
The coefficient of the influence of plants (IF_P_) on the number of organotrophic bacteria (Org), actinomycetes (Act), fungi (Fun), *Pseudomonas* sp. (Ps), *Arthrobacter* sp. (Art), and cellulolytic bacteria (Cel) in the soil contaminated with B, Zn^2+^, and B + Zn^2+^—PCA method; S—*Sorghum Moench*; P—*Panicum virgatum*; C—uncontaminated soil; B—bisphenol A; Zn—ion zinc (Zn^2+^).

Based on the influence index of the plants (IF_P_), both sorghum (S) and switchgrass (P), the extent of their effect on the microbiological activity of soil was determined (Figure 7). Following the PCA generated values of coordinates of cases and distances between them, it was concluded that the cultivation of sorghum (S) had a positive effect on the counts of organotrophic bacteria, actinomycetes, and the bacteria Pseudomonas sp. and Arthrobacter sp. in soil polluted with B + Zn^2+^, and on the counts of fungi and cellulolytic bacteria in soil under the pressure of combined toxicity and sown with switchgrass (Pv). 

The colony development (CD) and ecophysiological diversity (EP) indices were analyzed, taking into consideration the effect of soil contamination, sowing the soil with plants, and soil biostimulation with humic acid (Figures S1 and S2). The distribution of cases on the PCA map showed that the soil pollution with Zn^2+^ slightly inhibited the multiplication of organotrophic bacteria, regardless of whether the soil was biostimulated with humic acid (Figure S1). The adverse effect on the multiplication rate of this microbial group was also noted in the objects sown with sorghum (S) and polluted with the compilation of B + Zn^2+^. The application of the pollutants to soil did not significantly modify the multiplication of actinomycetes or fungi. In turn, the high CD values for fungi revealed the potential of this group for rapid multiplication. The PCA analysis also highlighted the fact that the combined toxicity of B + Zn^2+^ adversely affected the ecophysiological diversity (EP) of fungi, which, incidentally, scored the lowest EP values, regardless of the analyzed factors through the prism of which a given parameter was studied (Figure S2). However, the lowest EP value for organotrophic bacteria was noted in the control object polluted with bisphenol A, whereas organotrophic bacteria obtained the lowest EP in soil contaminated with bisphenol A and Zn^2+^ and sown with sorghum (S).

#### 2.2.2. Microorganisms Identified by the NGS Method

**Figure 8:**
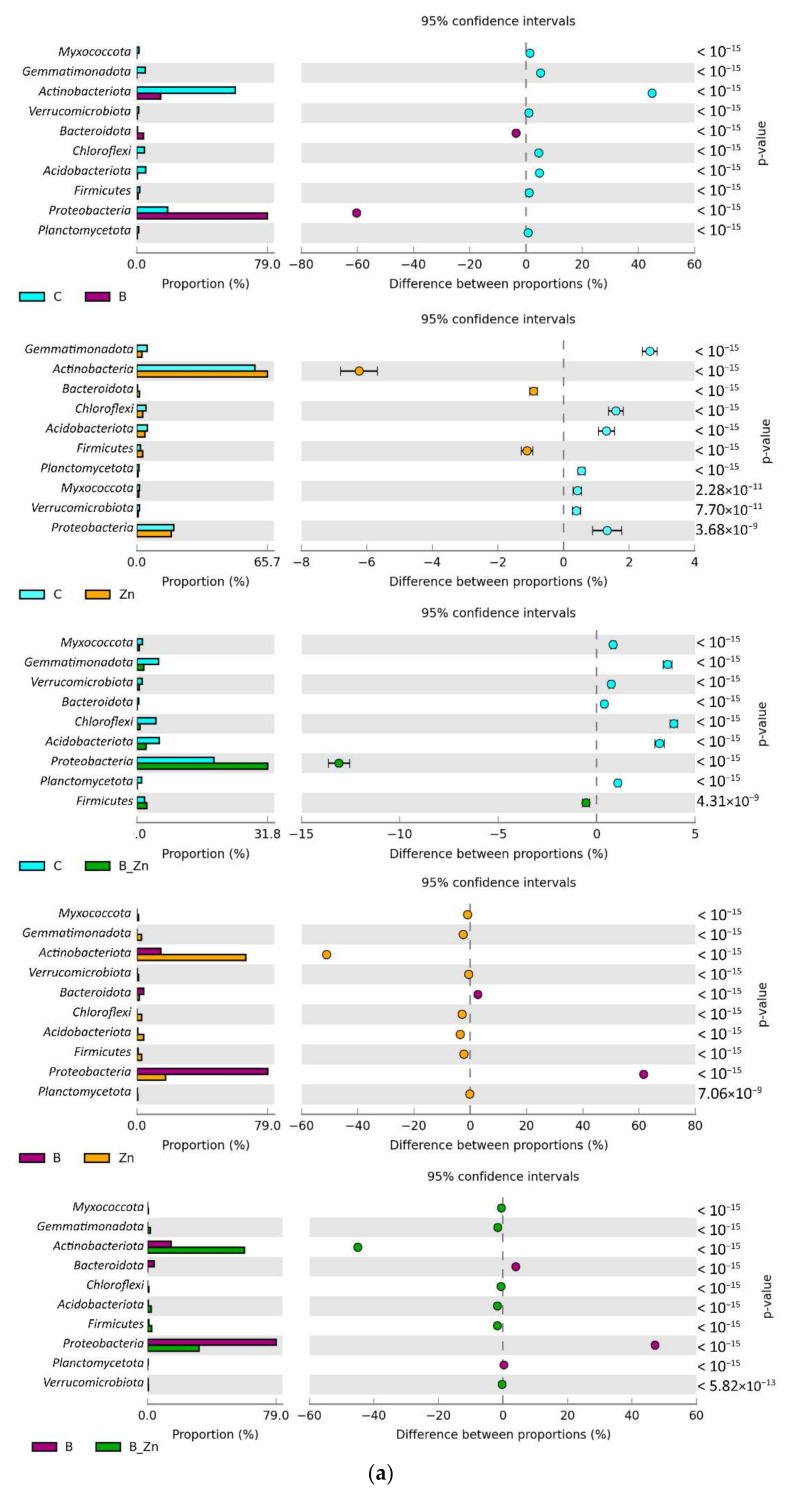

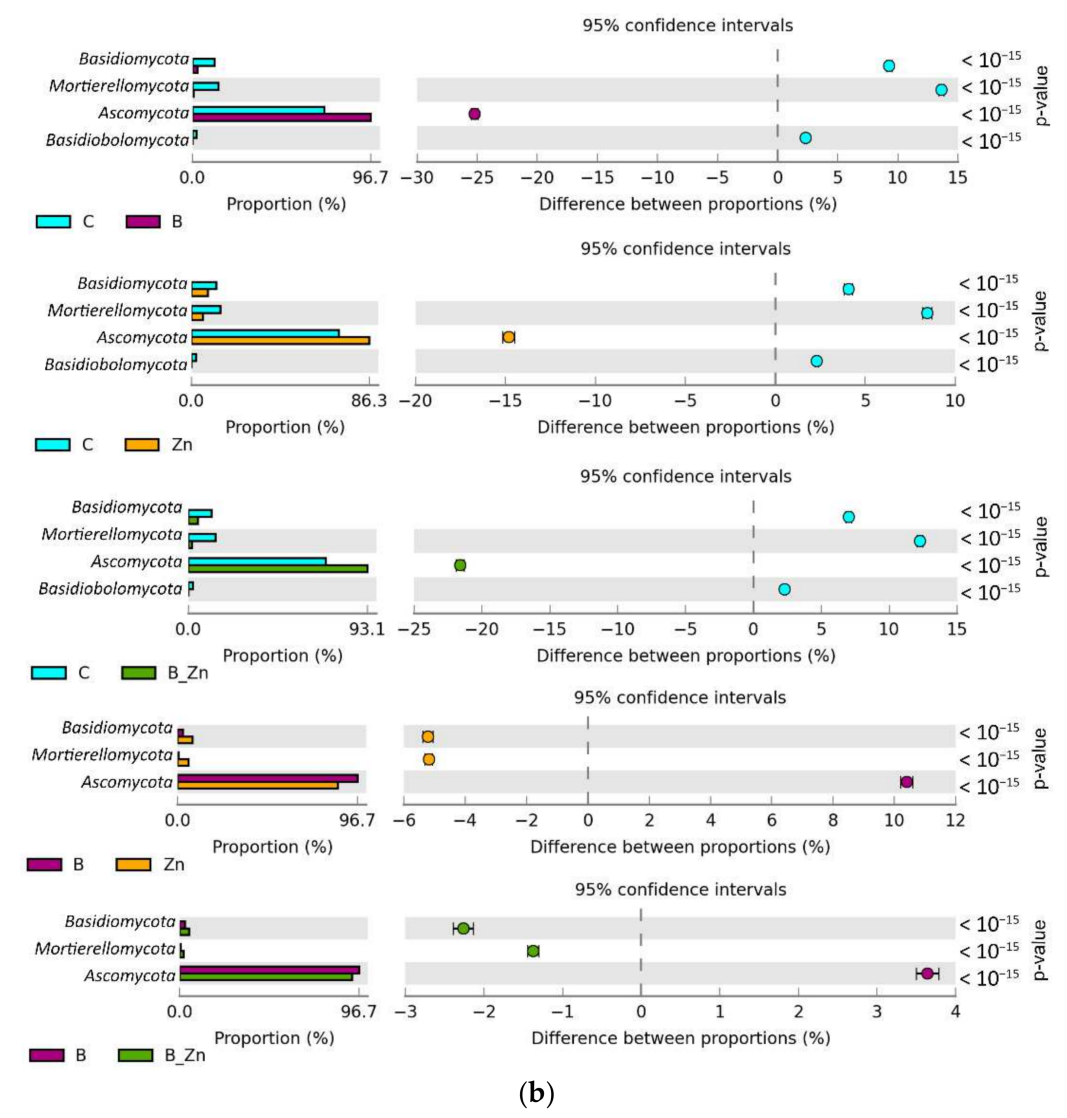
Relative abundance of the dominant types of bacteria (**a**) and fungi (**b**) in the soil, with a difference between proportions ≥1%; C—uncontaminated soil; B—bisphenol A; Zn—ion zinc (Zn^2+^).

The highest OTU abundance among the distinguished phyla in all soil samples was achieved by *Actinobacteriota* and *Proteobacteria* (Figure 8a). The OTU richness of *Actinobacteriota* in soil unpolluted with the xenobiotics was 34,574, composing 59% of all bacteria. It represented 14.57% of all bacteria in the bisphenol A polluted soil, 60% in the soil contaminated with B + Zn^2+^, and as much as 59% in the soil exposed to the pressure of Zn^2+^. In turn, *Proteobacteria* dominated in the bisphenol A polluted soil. The OTU value for the phylum *Proteobacteria* in this object reached 58,592, which made up as much as 79% of all bacteria. Among the objects contaminated with the xenobiotics, the lowest diversity of phyla was determined in the soil polluted with bisphenol A, in which only the phyla Proteobacteria, *Actinobacteriota*, and *Bacteroidota* were identified. According to OTU values counted at the level of phyla, molds were represented mainly by *Ascomycota*, *Mortierellomycota*, and *Basidiomycota* (Figure 8b). *Ascomycota* represented 97% of all fungi in B polluted soil. However, the application of this phenolic compound to soil decreased the OTUs of *Ascomycota* from 201,057 to 131,737 OTUs in soil exposed to the combined toxicity of B + Zn^2+^, whereas the soil contamination of Zn^2+^ decreased the richness of this phylum to 130,974. The smallest number of OTUs of *Ascomycota*, equaling 71,637, was determined in the control soil. The second most abundant microorganisms were the fungi *Basidiomycota,* present in all objects. The highest abundance of OTUs of this phylum was determined in soil contaminated with Zn^2+^, where *Basidiomycota* represented 8% of all fungi, and their abundance was comparable to that in unpolluted soil. The least numerous OTUs (3743) were determined in soil exposed to bisphenol A. Both the xenobiotics and their combinations eliminated the phylum *Basidiobolomycota* from the soil, which were identified in the control soil.

**Figure 9:**
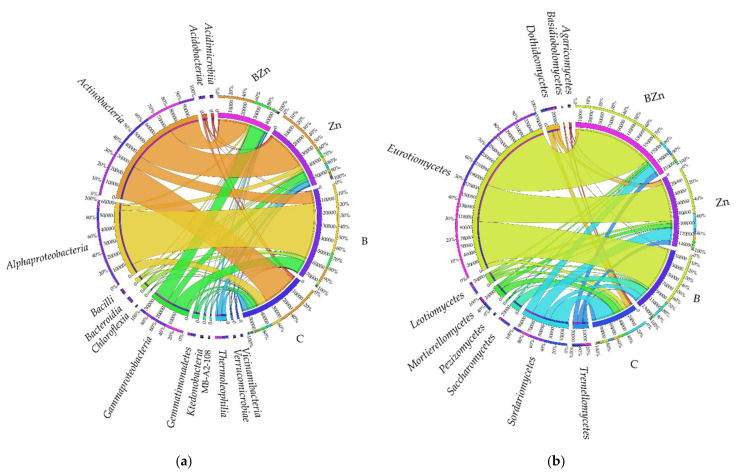
The relative abundance of dominant classes of bacteria (**a**) and fungi (**b**) in soil with a difference between proportions ≥1%; C—uncontaminated soil; B—bisphenol A; Zn—ion zinc (Zn^2+^).

According to the OTU values higher than 1% of assigned sequences, determined at the level of classes, *Actinobacteria*, *Alphaproteobacteria*, and *Gammaproteobacteria* proved to be representative among the analyzed 14 classes (Figure 9a). In three objects, control (29,339 OTUs), Zn^2+^ polluted (33,533 OTUs), and B + Zn^2+^ polluted soil (24,589 OTUs), the highest values were achieved for *Actinobacteria*, whereas the highest OTU value in soil contaminated with bisphenol A was obtained by *Alphaproteobacteria* (47,654 OTUs). It is worth noting that both bisphenol A and Zn^2+^ generated high abundance of the class *Bacteroida*, whose contribution in the soil exposed to the combined toxicity B + Zn^2+^ did not exceed 1%. Changes in the structure of fungi at the phylum level were reflected in their diversity at the class level (Figure 9b). In unpolluted soil samples, fungi were represented by 10 classes, dominated by *Sordariomycetes* (39,702 OTU), *Mortierellomycota* (142,220 OTU), and *Dothideomycetes* (7027 OTU). In this pool, the classes that most abundantly represented microorganisms resistant to bisphenol A pressure proved to be *Eurotiomycetes*, which represented 70% of all fungi, *Saccharomycetes* (13%) and *Sordariomycetes* (10%). However, it needs to be highlighted that the applied xenobiotics and their compilation contributed to a significant moderation of the richness of fungi from the class *Saccharomycetes*, which in unpolluted soil, made up 41.60% of all fungi. Bisphenol A decreased this share by 31%, Zn^2+^ by 23%, and B + Zn^2+^ by 33%. A positive effect of the particular contamination variants was observed on the class *Eurotiomycetes*. An increase in the share of this class of fungi, by 60%, 47%, and 68% relative to the controls, was noted.

**Figure 10:**
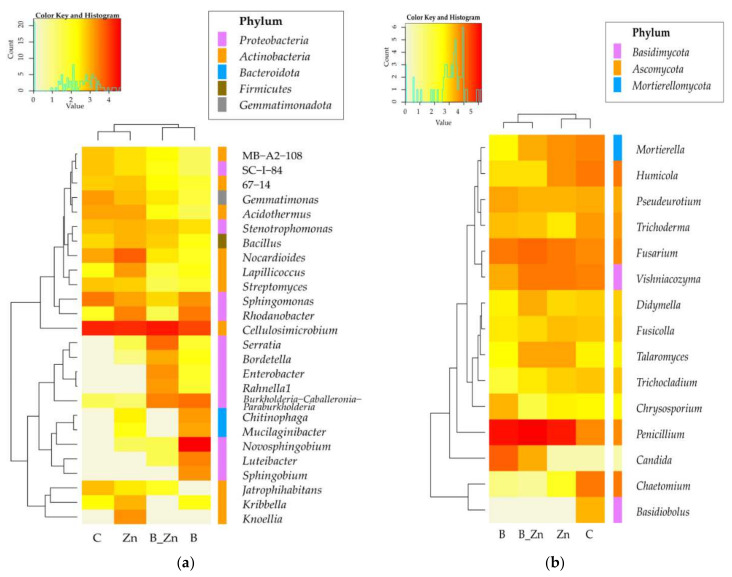
Heat map of the dominant genus types of (**a**) bacteria and (**b**) fungi in the soil. Data in the figures are represented by decimal logarithms, with a difference between proportions ≥1%; C—uncontaminated soil; B—bisphenol A; Zn—ion zinc (Zn^2+^).

**Figure 11:**
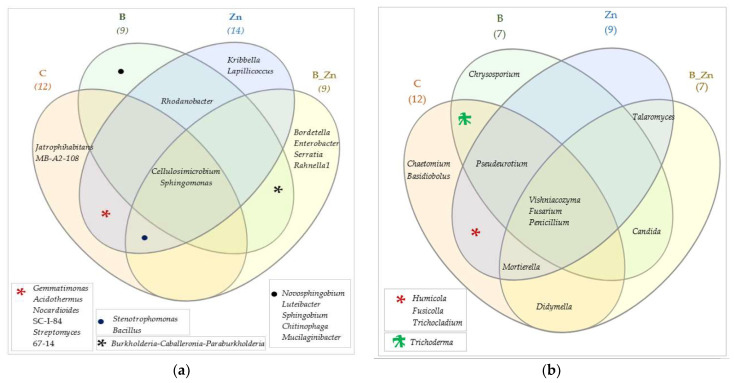
Venn diagram showing unique and common types of bacteria (**a**) and fungi (**b**), based on OTU ≥1%; C—uncontaminated soil; B—bisphenol A; Zn—ion zinc (Zn^2+^).

Changes in the structure of bacteria at the level of classes led to the regrouping at the level of genera (Figure 10a). In samples of the soil polluted with bisphenol A, the microbiome profile on this taxonomic level was shaped by the genus *Novosphingobium*, assigned to the class *Alphaproteobacteria,* phylum *Proteobacteria*, which made up 61% of all bacteria. The soil contaminated with Zn^2+^ or with B + Zn^2+^ generated the highest share of *Cellulosimicrobium*, a representative of the class *Actinobacteria*, phylum *Actinobacteriota*, which composed 45% and 61% of all bacteria in the two types of soil, respectively. The combined pollution with bisphenol A and Zn^2+^ stimulated the abundance of bacteria of the genus *Burkholderia-Caballeronia-Paraburkholderia*, while the pollution of soil with Zn^2+^ eliminated this bacterium from the pool of microorganisms, as well as inhibited the counts of *Cellulosimicrobium*. Consequences of an escalating negative impact of the combined contamination of B + Zn^2+^, in comparison with the negative effects of the xenobiotics applied separately, were observed against *Rhodanobacter* and *Sphingomonas*. Unique taxa of bacteria characteristic for the designed objects were identified (Figure 11a). These were: *Novosphingobium*, *Luteibacter, Sphingobium, Chitinophaga*, and *Mucilagnibacter* in soil polluted with B; *Lapilicoccus* and *Kribella* in the variants with Zn^2+^, and *Serratia, Enterobacter, Rahnella1,* and *Bordetella* when both xenobiotics were applied to soil.

Consistent with the results for the higher taxonomic levels, the differentiation of 15 identified types of fungi in the OTUs was demonstrated. Regardless of the type of contamination, *Penicillium, Fusarium,* and *Vishniacozyma* were determined to be the dominant forms(Figure 10b).

The highest OTUs of *Penicillium*, from the family *Aspergillaceae*, order *Eurotiales,* and class *Eurotiomycetes*, were found in soil polluted with B + Zn^2+^ (161,828 OTU). B decreased the number of OTUs of *Penicillium* by 44%, and Zn^2+^—by 53%. In unpolluted soil, fungi of the genus *Penicillium* composed no more that 10% of all identified genera of molds whose content in soil exceeded 1%. Under the pressure of bisphenol A, the OTUs of *Vishniacozyma* decreased as well, from 67,180 in unpolluted samples to 2900 OTU. Finally, the application of Zn^2+^, also in combination with the phenolic compound, induced an increase in the number of OTUs of this genus by 17% and 31%, respectively (Figure 11b).

Based on the individual values of the Shannon–Wiener (H) and Simpson (D) indices, it was demonstrated that bisphenol A, both when applied alone and in combination with Zn^2+^, decreased the diversity of bacteria and fungi to a larger extent than Zn^2+^ applied to soil alone (Table S3).

### 2.3. Response of Plants to Soil Contamination with Bisphenol A and Zinc

**Figure 12:**
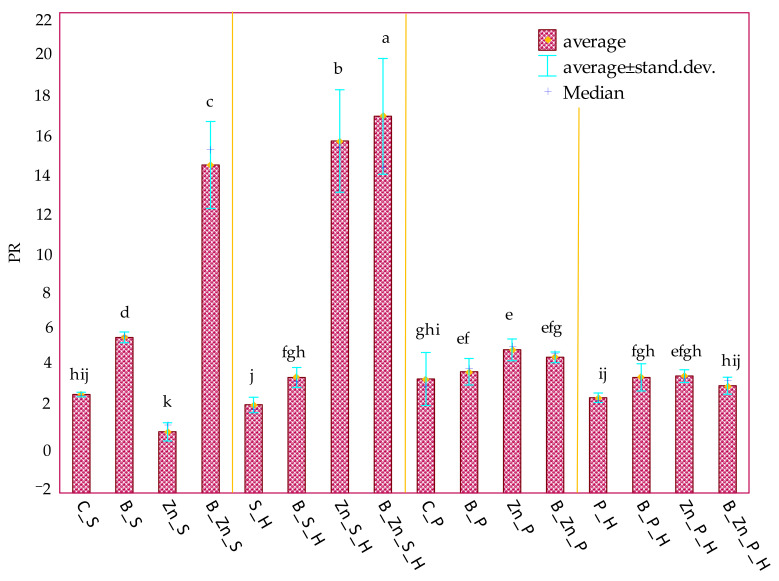
The ratio of the mass of the aerial parts of the plants to the roots (PR) in the soil contaminated with B, Zn^2+^, B + Zn^2+^; C—uncontaminated soil; S—*Sorghum Moench*; P—*Panicum virgatum*; H—humic acid; B—bisphenol A; Zn—ion zinc (Zn^2+^). Homogeneous groups denoted with letters (a–k) were calculated for all PR ratio values.

The response of sorghum (S) and switchgrass (P) to the soil contamination with the xenobiotics was traced in the experiment (Table S4). Bisphenol A added to soil least disturbed the yields of these plants, which is confirmed by the highest values of the tolerance index of the aerial biomass of sorghum (TI = 66.094) and of switchgrass (P) (TI = 54.680) (Table 1). However, the TI values determined for the roots of these plants were lower by 47% and 21%, respectively, which indicates a stronger negative effect of this phenolic on the development of the root system of both sorghum (S) and switchgrass (P). Significant disturbances of the growth and development of sorghum (S), in both the aerial parts and the roots, were observed under the pressure of the combined bisphenol A and Zn^2+^ toxicity. In turn, among the pool of objects polluted with Zn^2+^, the TI values obtained were higher than in objects exposed to the combined toxicity of zinc and bisphenol A by as much as 83%. The tolerance of switchgrass (P) to soil pollution with B + Zn^2+^ was higher than that of sorghum (S). Moreover, the application of humic acid contributed to the stimulated growth of only sorghum (S) in objects contaminated with bisphenol A, namely by 13% of the plant’s aerial parts and by 23% of its roots. The observed tendencies correspond with the values representing the ratio of aerial biomass to roots (PR) for both crop species (Figure 12), where the highest PR values were determined in the pots polluted with B + Zn^2+^ and sown with sorghum (S). 

Another parameter tested in this study was the relative content of chlorophyll, expressed with the SPAD leaf greenness (Table S5). It is worth emphasizing that—regardless of the type of pollution applied to soil—there was a rise in the SPAD values determined for sorghum (S), oscillating between 30% and 37%. The enrichment of soil with humic acid increased the relative chlorophyll content of the leaves of switchgrass (S), both in the controls and in the objects polluted with the xenobiotics.

## 3. Discussion

### 3.1. Soil Enzymes

The reaction of soil enzymes observed in this study being contrary to the response of microorganisms to bisphenol A brought to light the complexity of forms in which they occur in soil. Although Deh are tightly linked with oxidation-reduction processes and depend on the activity of living microorganisms, pH is a significant factor moderating Deh in the soil environment. An increase in this parameter destroys ionic and hydrogen bonds in the active center of the enzyme [39,40]. Studies carried out by Siczek et al. [41] and Zaborowska et al. [42] also lend credence to the research results achieved in our experiment. The toxicity of bisphenol A could be linked to the fact that one of Deh cofactors is pyrroloquinoline quinol (PPQ), which is responsible for the transport of electrons from the substrate to ubiquinone during the process of oxidation [43]. In turn, quinones are considered to be toxic intermediate metabolites of phenols and inhibitors of the enzymatic activity of both dehydrogenases and urease [44]. The inhibitory effect of bisphenol A on the activity of Ure might be linked to the inactivation of this enzyme, which relies on the formation of stable covalent adducts between the inhibitor and the enzyme’s functional groups [45].

The application of Zn^2+^ to soil caused the inhibition of the activity of all soil enzymes. The most severe inhibition affected Deh and Pac. A similar response of enzymes, including Pal, Cat, and Ure, to the Zn^2+^ pressure in soil has been noted by numerous researchers [46,47,48,49,50]. Undoubtedly, Zn^2+^ ions are considered to be inhibitors of enzymatic activity due to their relatively strong effect on active sites of enzymes with the catalytic dyads Cys-Cys, His-His, Glu(Asp)-Glu(Asp), Cys-His, Glu(Asp)-His, Cys-Glu(Asp), or triads with three of these amino acids, which are typical Zn^2+^ binding ligands [51]. Interestingly, B only slightly alleviated the negative effect of Zn^2+^ on the activity of Glu. This is probably due to the fact that the activity of Glu is closely correlated with the bioavailability of carbon, of which B is a source, and is linked with phenolic ring hydroxylation and the meta-cleavage pathway [52,53]. Besides, carboxyl and phenolic groups are sites of zinc complexation, which may reduce its availability and mobility in soil [54]. The extent of the positive effect produced by humic acid on the biochemical activity of soil, and indirectly on the soil condition, did not bring about the expected, spectacular effects because humic acid reduced the bioavailability of metals via strong affinity, as well as the ability of forming stable chelates with metal ions. Carboxyl and phenol-OH groups are responsible for this process [55,56]. In turn, considering its constant stability (8,1), zinc has a lower affinity for humic acid than Pb (14,8) or Cu (13,3) [57]. According to Lin et al. [58], manganese oxides and hydroxides participate in the effective degradation of organic pollutants, including bisphenols, while humic acids can inhibit the process of sorption of B with active hydroxyl groups by competing for binding sites.

### 3.2. Number and Variety of Bacteria and Fungi

The response of the analyzed groups of microorganisms, both bacteria and fungi, to the contamination of soil with bisphenol A is fully justified because the biochemical degradation of complex phenolic compounds leads to an increase in the abundance of subpopulations biodegrading B [59]. The response of the microbiome, especially Org, Act, Fun, Art, and Ps, is dictated by the participation of microorganisms in catabolic pathways of the aerobic and anaerobic degradation of phenolic compounds, which have been thoroughly analyzed by many researchers [60,61,62]. The key, triggering stage is the occurrence of hydroxyl groups in the environment, which is synonymous with the presence of oxygen as a co-substrate [63]. Two basic bisphenol A biodegradation mechanisms have been described, including the rearrangement of the skeleton of the aliphatic methyl group [60] and the hydroxylation of one or two phenyl rings, followed by cleavage of the aromatic ring [62]. The prevalent amounts of isolates identified in the environment exposed to the contamination with phenolic compounds were still composed of species of the genus *Pseudomonas* [64], which explains the increase in the abundance of *Pseudomonas* sp. by as much as 47%, in relation to the controls observed in our experiment. The fact that B generated an increase in the count of organotrophic bacteria by as much as seven-fold, coinciding with the lack of any increase in the ecophysiological diversity EP of this group of microorganisms, could be explained by the abundant representation of this group by *Bacillus* sp., which are distinguished by bioremediation potential towards B [65]. Admittedly, in view of the lowest CD values for actinomycetes, they can be considered as slow growing microorganisms [66], although the induction of a rise in their abundance in the presence of 1000 mg bisphenol A kg^−1^ d.m. of soil proves that they aspire to the role of microorganisms, effectively biodegrading bisphenols. This is confirmed by their enzymatic potential, represented by proteases, catalases, chitinases, amylases, and lectinases [61]. A large resource of enzymes catalyzing the degradation of bisphenol A also constitutes a response to the increase in the abundance of mold fungi exposed to this phenolic compound. The pool of these enzymes includes: lignin peroxidase, laccase, manganese-dependent peroxidase, triphenylmethane reductase, and polyketide synthase (PKS), which belongs to the group of cytochrome P450 enzymes [67,68]. Interestingly, the application of Zn^2+^ to soil caused an increase in the abundance of only organotrophic bacteria. It was expected that the response of the analyzed groups of microorganisms exposed to Zn^2+^ would be more varied. On the one hand, we know the different strategies of adaptation and tolerance of microorganisms to soil contaminated with zinc, which include cumulative mechanisms involving the synthesis of metallothionines, or the extracellular production of siderophores and polysaccharides [69,70]. On the other hand, reviewing the literature concerning the toxicity of heavy metals, it was expected that Zn^2+^ would have an inhibitory effect on the analyzed groups of microorganisms. These expectations were based on such effects as irreversible damage to cellular membrane integrity, inactivation or oxidation of cell enzymes by heavy metals absorbed in the cytoplasm, protein denaturation, damage to genetic material, or inhibition of transcription [71,72]. The results of this research have been confirmed by the observations of other researchers [47,49,50].

However, the inhibitory effect of the combined B + Zn^2+^ toxicity on organotrophic bacteria raises some controversy. It is worth bearing in mind that many pollutants that present jointly in the environment can exert not only additive or synergistic, but also antagonistic effects on one another [73]. Thus, bisphenol A may cause damage to the mitochondria, nucleus, and endoplasmic reticulum, as well as induce the peroxidation of cellular membrane lipids [74,75]. In turn, Zn^2+^ would then be responsible for the retardation of metabolic functions or modulations in genetic material [76].

The richness of soil phylotypes is usually generated by representatives of *Alphaproteobacteria*, *Betaproteobacteria*, *Actinobacteria*, and *Acidobacteria* [77]. Saalam and Varma [78] also emphasize the importance of the phylum *Proteobacteria*. The microbiome found in soils submitted to the pressure of xenobiotics, including B and Zn^2+^, is mainly represented by the phyla *Proteobacteria* and *Actinobacteriota*. The results obtained in this research correspond well with the observations of Zaborowska et al. [42] The observed tendencies arise from the fact that *Proteobacteria* represent both slow growing oligotrophic taxa and more rapidly growing copiotrophic taxa, with different metabolic properties and the highest diversity and abundance of genes resistant to xenobiotics, including heavy metals [79,80]. The diversity of the soil microbiome also corresponds to the mechanisms observed among representatives of the phylum *Proteobacteria*. For this reason, the dominance of bacteria from the genera *Sphingobium* and *Novosphingobium* determined in soil polluted with B and examined in this study may have been expected. A species of bacteria called *Sphingobium baderi*, resistant to B, was determined in the same objects. According to Zhang’a et al. [81], bacteria from the genus *Novosphingobium* can co-metabolize, or use B as a source of carbon. Ogata et al. [62] point to the bioremediation potential of *Sphingobium* sp. The identification of bacteria from the genus *Serratia* in objects contaminated with B + Zn^2+^ can be explained by the fact that they are equipped with class B metallo-*β*-lactamases [MBL], which bind zinc ions [82]. The exposure of the xenobiotics also affected the biodiversity of fungi. Regardless of the applied pollution variant, molds of the genera *Penicillium*, *Fusarium*, and *Vishniacozyma* were detected in all the objects, and the dominant molds in all the objects were *Penicillium elleniae, Penicillium subrubescens*, and *Penicillium javanicum.* The escalating abundance of OTUs of *Penicillium* may have been expected because, as reported by Al-Zaban et al. [83], these fungi are equipped with both laccases and peroxidases, catalyzing the degradation of phenolic compounds.

### 3.3. The Response of Sorghum and Millet to B, B + Zn^2+^ and Zn^2+^ Pressure

The low tolerance of sorghum and switchgrass to the xenobiotics added to soil is confirmed in many scientific papers [84,85]. There is no doubt that both bisphenol A and Zn^2+^ cause disturbances in the functioning of cellular organelles and in the uptake of nutrients [84,86]. The inhibition of the growth of roots of both tested plants is a confirmed, undesirable effect of soil contaminated with bisphenol A. Wang et al. [36] suggest that this effect is closely connected with the log_Kow_ of bisphenol A, which is A = 3.40, and which results to poor translocation of this phenolic in plants, hence favoring its accumulation in roots with a typically high fat content. This notwithstanding, the tolerance of both sorghum (*Sorghum Moench*) and switchgrass (*Panicum virgatum*), evaluated through the prism of plant yields, was the highest in soil polluted with bisphenol A, in comparison with the objects contaminated with the other xenobiotics. This probably arises from the fact that plant cells are able to generate metabolites of bisphenol A quite soon after absorbing this chemical, owing to glycosylation, selective hydroxylation, glycosylation, or redox reactions [87,88]. Of note is the fact that the application of bisphenol A to soil induced an increase in the chlorophyll content of the leaves of the crops, while Kim et al. [89] report that exposure to 500 mg B kg^−1^ d.m. in soil not only decelerated the rate of photosynthesis, but also contributed to a reduction in the size of the stomata and the content of chlorophyll a and chlorophyll b in the leaves. It also interfered with the fluorescence of this pigment. Nevertheless, Li et al. [90] implicate that the suppression of photosynthesis is not induced by bisphenol A. They observed that this phenolic compound inhibited carbon assimilation, leading to surpluses of electrons responsible for the activation of the photosynthetic system reaction centers, which in turn, could be a response to the positive effect of humic acid applied in that study on the mentioned parameter.

Reasons for the negative response of crops to zinc could be sought in the toxic effect of this heavy metal, resulting in the denaturation of proteins and DNA damage, which eventually leads to the death of cells [91]. The explanation for the lower yields of plants grown in soil contaminated with Zn^2+^ or B + Zn^2+^ than in soil exposed to B could only be found in the generally accepted claim that most of this heavy metal absorbed by a plant is transported symplastically, or through the apoplast from the root to the xylem [92]. It is also known that zinc is an essential element in many biochemical processes, such as the metabolism of auxins, lipids, and fatty acids, or the activation of enzymes or the production of chlorophyll [93]. However, in a dose over 700 mg Zn kg^−1^ d.w. in leaves, the symptoms of the toxicity of this metal emerge, such as leaf chlorosis, growth retardation, and oxidative stress [94]. According to Leskova et al. [95], this is largely connected with the secondary deficit of Fe or Mn, due to the competition between zinc and other metals for transport and protein binding sites. It needs to be stressed, however, that detoxication of zinc in the rhizosphere can involve the mechanisms through which roots release nicotianamine (NA) [96], oxalate, deoxymugineic acid (DMA) [97], or—in the case of sorghum—citrate [98].

## 4. Materials and Methods

### 4.1. Characteristics of the Soil

Soil was sampled from an area in the northern part of the Olsztyn Lake District (NE Poland, 53.72° N, 20.42° E), which lies in the geographical area called the East European Lowland. Most of the region’s terrain is composed of glacial till, sandy eluvia of glacial till, and sands with glacier boulders. These are accompanied by sands and gravels. The soil selected for the research was a Eutric Cambisol, sampled from a depth of 0–20 cm. It was loamy sand with the following textural composition: clay (d ≤ 0.002 mm)—3.71%, silt (0.05 ≥ d > 0.002 mm)—32.68%, sand (2.00 ≥ d > 0.05 mm)—63.61%. The characteristics of the soil material, including selected physicochemical properties, as well as biochemical and microbiological parameters, are shown in Table S6. The pot experiment was conducted in a greenhouse at the University of Warmia and Mazury in Olsztyn.

### 4.2. Design of the Experiment

To determine the effect of bisphenol A and zinc on the microbiological and biochemical activity of soil, as well as differences in the toxicity of these chemicals, the design of the experiment included objects with unsown soil and with soil sown, with two crop species. The response of sorghum (*Sorghum Moench*) and switchgrass (*Panicum virgatum*) to the application of the xenobiotics to soil was traced in the experiment, conducted under monitored conditions. The experiment was carried out in four treatments. Before the experiment was set up, the soil samples were mixed with mineral fertilizers, in amounts to satisfy the nutritional needs of the grown plants, that is, in the following doses expressed in pure element quantities: N—150, P—30, and K—135 mg kg^−1^ of soil. Each batch of 3.5 kg of soil packed in polyethylene pots was contaminated with the tested xenobiotics in the following amounts: 0 and 1000 mg B, as well as 1000 mg Zn^2+^ kg^−1^ d.m. of soil, and the compilation of B and Zn^2+^, each in doses of 1000 mg kg^−1^ d.m. of soil. Considering the poor solubility of bisphenols in water, prior to mixing with soil, the chemical was dissolved in ethanol, in the 3:1 ratio (ethanol:bisphenol). Zinc was added to soil in the form of ZnCl_2_, dissolved in deionized water. An analysis of the response of the soil microbiome to the increasing contamination with the xenobiotics was made on the 50th day of the experiment, in three replications, after harvesting the crops. The biostimulating potential of humic acid, alleviating the undesirable effect of B and Zn^2+^ on the condition of the soil, was determined owing to the application of a substance composed of humic acid (90%), potassium, sulfur, and macronutrients in trace amounts (Lignohumat super; Agrarius, Poland). This humic preparation was applied in doses of 0 and 4 g kg^−1^ d.m. of soil.

### 4.3. Plants

The choice of plants was dictated by the growing popularity of these species in the global arena [99,100]. Importantly, sorghum *Sorghum Moench* (S) raises methane yield by 2.8–7.7% in liquid anaerobic digestion [101]. In turn, switchgrass (P) is a perennial, fast-growing crop, which is seen as a promising resource of lignocellulose for the production of biofuels [102]. The experiment was conducted in two series, with 10 seeds of sorghum *Sorghum Moench* (S) sown in each pot in one series, and 10 seeds of switchgrass (*Panicum virgatum*) (P) per pot in the other series. At phase BBCH 10, the plants were thinned, leaving 3 sorghum plants and 4 switchgrass plants per pot. The SPAD (Soil and Plant Analysis Development) leaf greenness index was measured for both plants. The mean SPAD value was determined based on the readings on 5 leaves per plant before harvest. The determinations were made with a SPAD 502 Chlorophyll Meter 2900P. At phase BBCH 50, the plants were harvested. The aerial parts and roots, once weighed, were dried for 5 days at a temperature of 60 °C.

### 4.4. Physicochemical Analyses

The establishment of the experiment in a greenhouse was preceded by a series of analyses, where selected physicochemical properties of soil sifted through a 2 mm mesh sieve were determined. All determinations were carried out using appropriate methods, namely: the textural composition of soil (aerometric method) [103], the soil pH in 1 mol KCl (potentiometric method) [104], hydrolytic acidity (HAC), exchangeable base cations (EBC) in mmol (+) kg⁻¹ (Kappen’s method) [105], and organic carbon content (Corg) in g kg^−1^ (TIURIN’s method) [106]. Other determinations included the content of total nitrogen with the Kjeldahl method (Buchi B-324 distiller, Buchi, Flawil, Switzerland) [107], available phosphorus (P_available_) (Jenway 6705 UV/VIS spectrophotometer (Jenway LTD, Staffordshire, UK),and potassium (K_available_) (Jenway PFP 7 flame photometer (Jenway LTD, Staffordshire, UK) with the Egner–Riehm method [108], and magnesium (Mg_available_) by atomic absorption spectrometry (atomic absorption spectrophotometer GBC 932AA (GBC Scientific Equipment, Braeside, Australia) [109]. The HAC and EBC values served to determine the soil’s cation exchange capacity (CEC), expressed in mmol (+) kg⁻¹, and base saturation (BS) in %.

### 4.5. Biochemical Analyzes

The activity of seven enzymes in the soil was determined: dehydrogenases (Deh), catalase (Cat), urease (Ure), *β*-glucosidase (Glu), arylsulfatase (Aryl), acid phosphatase (Pac), and alkaline phosphatase (Pal), considered to be reliable indicators of fertility. The units used to express the values of activity of individual enzymes, the substrates used to determine their activity, and the wavelengths at which they were determined are presented in Table S7 [110,111]. The activity of the enzymes, with the exception of catalase, was determined using a Perkin-Elmer Lambda 25 spectrophotometer (Woburn, MA, USA). The catalase sensitivity to the pressure of the applied xenobiotics was determined using the residual hydrogen peroxide titration method. Based on the activity of all enzymes, the soil fertility biochemical index (BA_21_) was calculated [112]. The enzyme activity determination was performed in triplicate. Determinations of the activity of enzymes were made in three replicates.

### 4.6. Microbiological Analyses

#### 4.6.1. Determination of Counts of Soil Microorganisms

Counts of six groups of microorganisms were determined in each soil sample. These were: *Arthrobacter* sp. (Art) [113] and *Pseudomonas* sp. (Ps) [113], resistant to high doses of zinc and biodegrading bisphenol A [33,114], cellulolytic bacteria (Cel) [113] and organotrophic bacteria (Org) [115], actinomycetes (Act) [116], and fungi (Fun) [117], serving as the basis for the determination of two indicators: colony development (CD) [66] and ecophysiological diversity (EP) [118]. The determination of the abundance of all these groups of microorganisms was achieved by the serial dilution method, using the protocols described by Borowik et al. [119]. The colony forming units (cfu) of *Arthrobacter* sp. and *Pseudomonas* sp. were counted after 4 days of incubation, the cfu of cellulolytic bacteria after 14 days, and the cfu of organotrophic bacteria, actinomycetes, and fungi were counted for 10 consecutive days. Before all microbial groups were counted, they were incubated at a constant temperature of 28 °C. The number of colony forming units (cfu) was determined using a colony counter.

#### 4.6.2. DNA Isolation

Determination of the DNA with a Genomic Mini 647 AX Bacteria+ kit (A&A Biotechnology) was preceded by mechanical lysis with the use of mutanolysin and lysozyme to ensure the extraction and precipitation of the genomic DNA from 1 g of soil. The lysis was carried out in a FastPrep—24 apparatus using zirconia balls, and the DNA isolated from soil samples was determined using the colorimetric method.

#### 4.6.3. Metagenomic Analysis of Bacterial and Fungal Taxa

The bacterial region V3-V4 16S rDNA was amplified with the help of the following starters: 341F 5’ TCGTCGGCAGCGTCAGATGTGTATAAGAGACAGCCTACGGGNGGCWGCAG, 785R: 5’GTCTCGTGGGCTCGGAGATGTGTATAAGAGACAGGACT ACHVGGTATCTAATCC. The primers contained the Illumina adapter sequence (341F:TCGTCGGCAGCGTCAGATGTGTATAAGAGACAG, 785R:GTCTCGTGGGCTC GGAGATGTGTATAAGAGACAG), and a sequence specific for locus V3-V4 16S rRNA. The fungal ITS1 region was amplified with the following starters: ITS1FI2: 5’ TCGTCGGCAGCGTCAGATGTGTATAAGAGACAGGAACCWGCGGARGGATCA, 5,8S: 5’GTCT CGTGGGCTCGGAGATGTTATAAGGAGCGCTGCGTTCTTCATCG. The primers contained the Illumina adapter sequence (ITS1FI2:TCGTCGGCAGCGTCAGATGTGTATAAGAGACAG, 5,8S: GTCTCGTGGGCTCGGAGATGTGTATAAGAGACAG) and the sequence specific for the ITS1 locus. The next generation sequencing was carried out in an MiSeq apparatus by Illumina (Genomed S.A., Warsaw, Poland) in the mode of 2 × 300 PE (pair ends) in order to obtain the average number of 50,000 reads per sample. Based on the OTU values, the diversity of bacteria and fungi was determined, as illustrated with the Shannon–Wiener (H) and Simpson (D) indices [120].

#### 4.6.4. Statistical Analysis of Data and Methodology of Calculations

The data analysis software program Statistica 13.1 package TIBCO Software [121] was employed for the configuration of the research results. A multidimensional and explorative analysis PCA was used to illustrate the effect on the applied xenobiotics on values of the colony development coefficient (CD) and the ecophysiological diversity coefficient (EP), as well as the influence of the crops on the biochemical and microbiological activity of the soil. Tukey’s test at *P* = 0.01 was used to determine homogeneous variances between soil enzymes and microorganism groups and between the values of the: biochemical fertility index (BA_21_), plant tolerance index (TI), ratio of the mass of aerial parts of plants to roots (PR), and values of SPAD. The qualitative filtering of the reads and the classification to species were performed using the QIIME package according to the reference base GreenGenes (bacteria) and UNITE database (fungi). The data were visualized with the help of five software programs: Statistica 13.1 package TIBCO Software; with the interval confidence method Asymptotic with CC—STAMP 2.1.3.; with a two-sides test of statistical hypotheses; G-test (w/Yates’) + Fisher’s exact test; the thermal map was based on Rstudio v1.2.5033 R project, gplot v3.6.2 was used for the Venn diagram [122,123,124,125,126]. Using the Circos 0.68 software, the data were presented in a circular arrangement.

The reactions of enzymes and microorganisms were described on the basis of the coefficient of the influence of xenobiotics (IF_X_), humic acid (IF_H_), and plants (IF_P_) on the tested parameters, using the formula:

(1) $${\mathrm{IF}}_{X\_H\_P}=\frac{A_{X\_H\_P}}{A_{C}}$$

where:

IF_x_—The coefficient of the impact of soil contaminated with bisphenol A (B), Zn^2+^, B + Zn^2+^,
IF_H_—coefficient of soil biostimulation with humic acid,
IF_P_—plant influence coefficient,
IF_x_H_P_ < 1—inhibition of the activity of individual enzymes and the number of microorganisms; >1—stimulation of the activity of individual enzymes and the number of microorganisms,
A_x_—activity of individual enzymes and groups of microorganisms in soil contaminated with bisphenol A (B); Zn^2+^; B + Zn^2+^,
A_C_—activity of individual enzymes and the number of microorganisms in the control soil (uncontaminated soil).

The effect of the applied xenobiotics on the yield of the aerial part of the plants and roots was determined on the basis of the tolerance index (TI), which was calculated according to the formula:

(2) $$\mathrm{TI}\;=\;\frac{Y_{P}}{Y_{C}}\;\times \;100$$

where:

TI—tolerance index of plants (aerial part and roots for soil contaminated with bisphenol A (B), Zn^2+^, B + Zn^2+^ (TI < 100—inhibitory effect of xenobiotics; TI > 100—stimulating effect of xenobiotics),
Yp—yield of aerial parts and roots of plants in soil contaminated with xenobiotics,
YC—yield of aerial parts and roots of plants in the control soil, uncontaminated with xenobiotics.

The biochemical soil fertility index (BA_21_) was calculated using the formula described by Wyszkowska et al. [112]:

(3) $${\mathrm{BA}}_{21}\;=\;\mathrm{Deh}\;+\;\mathrm{Cat}\;+\;\mathrm{Pal}\;+\;\mathrm{Ure}\;+\;\mathrm{Glu}\;+\;\mathrm{Aryl}$$

where:

Deh—dehydrogenase, Cat—catalase, Pal—alkaline phosphatase, Pac—acid phosphatase, Ure—urease, Glu—*β*-glucosidase, and Aryl—arylsulfatase.

The CD and EP indices were calculated from the following two formulas:

(4) $$\mathrm{CD}\;=\;[\frac{N1}{1}\;+\;\frac{N2}{2}\;+\;\frac{N3}{3}\ldots \;\frac{N10}{10}]\;\cdot \;100$$

where:

N1, N2, N3… N10—sum of ratios of the colony numbers identified on each day (1, 2, 3, … 10) and the sum of all the colonies identified during the entire experiment

(5) $$\mathrm{EP}\;=\;-\Sigma \left(\mathrm{pi}\;\cdot \;\log\;\mathrm{pi}\right)$$

where:

pi—the number of microbial colonies on a given day divided by the number of all colonies.

The ratio of the mass of the aerial parts to the mass of the plant roots (PR) was also calculated using the formula:

(6) $$\mathrm{PR}\;=\;\frac{P}{R}$$

where:

PR—ratio of the mass of the aerial parts to the mass of the roots of plants, P—dry matter yield of aerial parts, and R—dry matter yield of roots.

## 5. Conclusions

The combined toxicity of bisphenol A and zinc had a stronger negative impact on the soil microbiome than either of the xenobiotics applied separately to soil. Bisphenol A did not participate in the complexation of zinc in soil. The response of the soil microbiome to the soil contaminated with bisphenol A (B), Zn^2+^, and B + Zn^2+^ was varied. Zn^2+^ and B + Zn^2+^ distorted the soil balance to a greater extent than bisphenol A, producing an inhibitory impact on the enzymatic and microbiological activity and on the diversity of microorganisms. The strongest inhibition was caused by the soil application of B + Zn^2+^. In both unpolluted soil and soil exposed to the xenobiotics, the dominance of representatives of the phyla *Actinobacteriota* and *Proteobacteria* among bacteria, and the phyla *Ascomycota* and *Basidiomycota* among mold fungi, was determined. The NSG analysis distinguished unique genera of bacteria characteristic for the particular types of soil contamination. These were *Novosphingobium*, *Luteibacter*, *Sphingobium*, *Chitinophaga*, and *Mucilagnibacter* for bisphenol A, *Lapilicoccus* and *Kribella* for Zn^2+^, and *Serratia*, *Enterobacter*, *Rahnella1*, and *Bordetella* for B + Zn^2+^. Among mold fungi, regardless of the type of contamination, three dominant genera were identified: *Penicillium*, *Fusarium*, and *Vishniacozyma*. Humic acid had a particularly stimulating effect on the activity of urease, counts of organotrophic bacteria, and *Pseudomonas* sp. However, it did not alleviate the negative effect of the xenobiotics on the growth and development of *Sorghum Moench* (S). This plant also proved to be much more sensitive to the soil contaminated with B + Zn^2+^ and Zn^2+^ than *Panicum virgatum* (P).

## Figures and Tables

**Table 1:** Plant tolerance index (TI) of *Sorghum Moench* (S) and *Panicum virgatum* (P) (aerial parts of plants and root) to soil contaminated with B, Zn^2+^, and B + Zn^2+^.

Object	TI of Aerial Parts of Plants	TI of Roots
	*Sorghum Moench* (S)	
B	66.094 ^a^	34.721 ^c^
Zn	9.620 ^g^	18.440 ^d^
BP_Zn	7.494 ^g^	2.330 ^h^
*Sorghum Moench* (S) with humic acid (H)		
B	51.301 ^d^	43.461 ^b^
Zn	2.705 ^h^	14.434 ^ef^
BP_Zn	4.946 ^h^	11.244 ^fg^
*Panicum virgatum* (P)		
B	54.680 ^c^	43.461 ^b^
Zn	20.015 ^e^	17.624 ^e^
B_Zn	17.777 ^e^	11.244 ^efg^
	*Panicum virgatum* (P) with humic acid (H)	
B	63.075 ^b^	56.439 ^a^
Zn	15.459 ^f^	10.775 ^g^
B_Zn	17.067 ^f^	14.520 ^efg^

B—bisphenol A; Zn—ion zinc (Zn^2+^). Homogeneous groups denoted with letters (a–h) were calculated separately for each part of the plants.

## Data Availability

Data are available by contacting the authors.

## Supplementary Materials

The following supporting information can be downloaded at https://www.mdpi.com/article/10.3390/ijms23115937/s1.

## Abbreviations

The following abbreviations are used in this manuscript:

B—bisphenol A; Zn^2+^—zinc ion; IF_X_—the coefficient of the impact of soil contamination with bisphenol A (B), Zn^2+^, B + Zn^2+^; IF_H_—coefficient of soil biostimulation with humic acid; IF_P—_plant influence coefficient; TI—tolerance index of plants; BA_21_—the biochemical soil fertility index; PR—ratio of the mass of aerial parts to mass of roots of plants; SPAD—the leaf greenness index; Org—organotrophic bacteria; Act—actinomycetes; Fun—fungi; Ps—*Pseudomonas* sp.; Art—*Arthrobacter* sp.; Cel—cellulolytic bacteria; Deh—dehydrogenases; Cat—catalase; Ure—urease; Pac—acid phosphatase; Pal—alkaline phosphatase; Aryl—arylsulfatase; Glu—*β*-glucosidase; CD—colony development index; EP—ecophysiological diversity index; H—Shannon–Wiener index; D—Simpson index; C_org_—total organic carbon; N_total_—total nitrogen; HAC—hydrolytic acidity; EBC—total exchangeable cations; CEC—total exchange capacity of soil; BS—basic cation saturation ratio in soil.
