# Supplementary material for: Effect of Separate and Combined Toxicity of Bisphenol A and Zinc on the Soil Microbiome
Source: Int J Mol Sci. 2022 May 25;23(11):5937. doi: 10.3390/ijms23115937 (PMC9180857; doi:10.3390/ijms23115937)
Supplement: Supplementary file 1 [file ijms-23-05937-s001.zip › ijms-1725311-supplementary.pdf]

International Journal of *Molecular Sciences*

MDPI

Supplementary

Effect of separate and combined toxicity of bisphenol A and zinc on the soil microbiome

Magdalena Zaborowska[1], Jadwiga Wyszkowska[1*], Agata Borowik[1], Jan Kucharski[1]

[1] Department of Soil Science and Microbiology, University of Warmia and Mazury in Olsztyn, Plac Łódzki 3, 10 - 727 Olsztyn, Poland; m.zaborowska@uwm.edu.pl; agata.borowik@uwm.edu.pl, jan.kucharski@uwm.edu.pl

*Correspondence: jadwiga.wyszkowska@uwm.edu.pl; https://orcid.org/0000-0002-2156-3780

Table S1. Enzymatic activity of soil contaminated with B, Zn^{2+} and B+Zn^{2+} per kg d.m. of soil h^{-1}

Objects	Deh (µmol TFF)	Cat (mol O_2)	Pac	Pal	Aryl	Glu	Ure (mmol N-NH_4)
			(mmol PN)				
			Unsown soil (U)				
C	4.050^{a}	0.353^{a}	4.232^{a}	0.636^{c}	0.326^{cd}	0.407^{c}	0.364^{a}
B	2.021^{b}	0.327^{b}	3.539^{b}	0.813^{b}	0.376^{bc}	0.503^{bc}	0.088^{d}
Zn	0.407^{cd}	0.294^{c}	1.765^{d}	0.584^{c}	0.279^{d}	0.227^{d}	0.101^{cd}
B_Zn	0.128^{de}	0.197^{e}	1.694^{de}	0.548^{c}	0.225^{e}	0.554^{ab}	0.014^{e}
			Unsown soil (U) with humic acid (H)				
C	1.700^{b}	0.329^{ab}	2.900^{c}	0.607^{c}	0.380^{b}	0.472^{bc}	0.234^{b}
B	1.707^{b}	0.338^{ab}	2.788^{c}	1.044^{a}	0.438^{a}	0.583^{ab}	0.141^{c}
Zn	0.670^{c}	0.267^{d}	1.547^{de}	0.675^{bc}	0.403^{ab}	0.444^{bc}	0.142^{c}
B_Zn	0.056^{e}	0.243^{d}	1.462^{e}	0.705^{b}	0.364^{bc}	0.660^{a}	0.062^{d}
			Soil sown with *Sorghum Moench* (S)				
C	8.356^{a}	0.324^{bc}	3.423^{a}	1.319^{a}	0.447^{bc}	0.402^{bc}	0.483^{a}
B	1.177^{de}	0.370^{a}	2.518^{b}	1.095^{b}	0.421^{cd}	0.274^{d}	0.160^{e}
Zn	1.888^{d}	0.315^{bc}	1.862^{c}	0.659^{d}	0.351^{e}	0.366^{c}	0.275^{c}
B_Zn	0.289^{e}	0.287^{cd}	1.890^{c}	0.748^{d}	0.353^{e}	0.509^{a}	0.058^{f}
			Soil sown with *Sorghum Moench* (S) with humic acid (H)				
C	7.282^{b}	0.347^{ab}	3.692^{a}	1.066^{b}	0.478^{ab}	0.417^{bc}	0.438^{b}
B	3.497^{c}	0.354^{ab}	2.458^{b}	1.046^{b}	0.499^{a}	0.449^{b}	0.266^{c}
Zn	1.694^{d}	0.322^{bc}	1.470^{c}	0.791^{cd}	0.449^{abc}	0.369^{c}	0.224^{d}
B_ Zn	0.314^{e}	0.268^{d}	1.378^{c}	0.992^{bc}	0.382^{de}	0.525^{a}	0.171^{e}
			Soil sown with *Panicum virgatum* (P)				
C	9.145^{a}	0.372^{a}	3.114^{b}	1,218^{a}	0,389^{de}	0,338^{de}	0,381^{b}
B	2.636^{d}	0.325^{bc}	2.078^{d}	0,919^{b}	0,481^{ab}	0,244^{f}	0,250^{d}
Zn	1.854^{e}	0.306^{c}	1.864^{dc}	0,576^{c}	0,347^{e}	0,345^{de}	0,255^{d}
B_Zn	0.330^{f}	0.289^{c}	1.834^{dc}	0.736^{bc}	0.337^{e}	0.503^{a}	0.214^{d}
			Soil sown with *Panicum virgatum* (P) with humic acid (H)				
C	6.315^{b}	0.375^{a}	3.664^{a}	0.898^{b}	0.487^{a}	0.477^{ab}	0.484^{a}
B	3.508^{c}	0.350^{ab}	2.492^{c}	0.818^{bc}	0.431^{abc}	0.324^{e}	0.321^{c}
Zn	1.451^{e}	0.301^{c}	1.614^{e}	0.797^{bc}	0.458^{ab}	0.392^{cd}	0.336^{bc}
B_Zn	0.137^{f}	0.295^{c}	1.961^{dc}	1.179^{a}	0.427^{cd}	0.416^{bc}	0.150^{e}

C- uncontaminated soil, U – unsown soil, B – bisphenol A, Zn - zinc ion (Zn^{2+}), Deh - dehydrogenases; Ure - urease, Pal – alkaline phosphatase, Pac – acid phosphatase, Aryl - arylsulfatase, Glu – *β*- glucosidase; C – uncontaminated soil, B – Bisphenol A, Zn – ion zinc (Zn^{2+}). Homogeneous groups denoted with letters (a-f) were calculated separately for the unsown objects, the soil sown with *Sorghum Moench*, and the soil sown with *Panicum virgatum*

Table S2. The number of microorganisms (jtk 10^n) in the soil contaminated with B, Zn^{2+} and B+Zn^{2+} per kg d.m. of soil

Objects	Org	Act	Ps	Art	Cel	Fun
			Unsown soil (U)			
C	31.755^{e}	94.167^{d}	145.592^{c}	42.632^{c}	14.479^{cd}	45.740^{b}
B	218.901^{a}	293.884^{a}	214.491^{b}	278.915^{a}	28.043^{b}	101.337^{a}
Zn	15.684^{e}	46.104^{f}	11.722^{e}	35.165^{c}	6.593^{e}	19.181^{c}
B_Zn	105.967^{b}	72.892^{e}	27.649^{de}	54.459^{c}	12.568^{c}	13.025^{c}
			Unsown soil (U) with humic acid (H)			
C	91.349^{b}	138.378^{c}	60.729^{d}	59.979^{c}	27.740^{b}	18.760^{c}
B	213.085^{a}	302.980^{a}	326.299^{a}	281.369^{a}	58.925^{a}	47.408^{b}
Zn	84.179^{bc}	106.326^{d}	69.127^{d}	125.618^{b}	9.663^{de}	34.411^{b}
B_Zn	49.559^{de}	184.125^{b}	291.181^{a}	168.464^{b}	17.427^{c}	106.594^{a}
			Soil sown with *Sorghum Moench* (S)			
C	62.559^{d}	106.958^{f}	75.881^{c}	92.141^{e}	42.586^{a}	13.990^{d}
B	132.708^{a}	133.799^{de}	309.724^{b}	148.860^{de}	20.008^{cd}	41.253^{c}
Zn	117.632^{ab}	231.044^{a}	527.639^{a}	396.116^{bc}	10.831^{d}	44.574^{c}
B_Zn	47.738^{d}	143.758^{cd}	294.258^{b}	62.998^{e}	34.290^{ab}	56.873^{b}
			Soil sown with *Sorghum Moench* with humic acid (H)			
C	103.775^{bc}	118.728^{ef}	102.004^{c}	139.891^{e}	47.359^{a}	16.692^{d}
B	129.893^{a}	160.592^{c}	558.961^{a}	435.604^{b}	35.465^{ab}	35.115^{c}
Zn	132.701^{a}	152.034^{c}	670.290^{a}	630.120^{a}	15.753^{cd}	62.403^{b}
B_Zn	88.842^{c}	201.014^{b}	202.328^{bc}	272.635^{cd}	28.123^{abc}	85.061^{a}
			Soil sown with *Panicum virgatum* (P)			
C	109.230^{bc}	117.962^{e}	97.427^{e}	115.498^{c}	16.500^{e}	12.964^{d}
B	102.283^{c}	129.724^{de}	151.515^{e}	108.112^{c}	11.048^{f}	56.818^{d}
Zn	131.632^{a}	154.173^{cd}	704.094^{a}	451.462^{a}	31.189^{b}	121.318^{a}
B_Zn	62.051^{c}	158.226^{c}	380.639^{c}	80.670^{c}	14.881^{e}	50.837^{bc}
			Soil sown with *Panicum virgatum* with humic acid (H)			
C	119.953^{ab}	180.091^{bc}	208.505^{de}	108.580^{c}	69.240^{a}	19.098^{d}
B	109.932^{bc}	228.195^{a}	325.562^{cd}	392.066^{a}	23.972^{d}	36.644^{c}
Zn	102.835^{c}	190.803^{b}	547.881^{b}	315.661^{ab}	25.190^{cd}	59.951^{b}
B_Zn	98.045^{c}	191.697^{b}	288.066^{cd}	223.620^{bc}	27.952^{bc}	116.363^{a}

Org – organotrophic bacteria, Act - actinomycetes, Fun - fungi, Ps - *Pseudomonas* sp., Art - *Arthrobacter* sp., Cel – cellulolytic bacteria, n- exponent 7 for Org, Act, Cel, 6 for Fun, Ps, Art. C – uncontaminated soil, B – Bisphenol A, Zn – ion zinc (Zn^{2+}). Homogeneous groups denoted with letters (a-f) were calculated separately for the unsown objects, the soil sown with *Sorghum Moench,* and the soil sown with *Panicum virgatum.*

Table S3. Shannon-Wiener and Simpson indices calculated from the abundance of operational taxonomic unit OTU

bacterial taxon	C	B	Zn	B_Zn
	Shannon-Wiener index (H′)			
phylum	1.44	0.73	1.29	1.06
class	2.05	1.15	1.83	1.42
order	3.02	1.53	2.79	1.93
family	3.40	1.62	3.27	2.24
genus	3.69	1.93	3.69	2.39
	Simpson index (D)			
phylum	0.61	0.35	0.55	0.55
class	0.72	0.54	0.66	0.62
order	0.89	0.60	0.84	0.69
family	0.98	0.85	0.94	0.88
genus	0.88	0.66	0.90	0.72

mold fungus taxon	C	B	Zn	B_Zn
	Shannon-Wiener index (H′)			
phylum	1.14	0.26	0.68	0.41
class	1.98	1.15	1.61	1.08
order	1.94	1.34	1.66	1.26
family	1.99	1.38	1.72	1.31
genus	2.07	1.39	1.73	1.33
	Simpson index (D)			
phylum	0.54	0.10	0.31	0.18
class	0.81	0.51	0.69	0.43
order	0.72	0.63	0.68	0.61
family	0.72	0.64	0.68	0.62
genus	0.71	0.64	0.68	0.62

C – uncontaminated soil, B – Bisphenol A, Zn – ion zinc (Zn^{2+})

Tabela S4. Dry matter yield of the aerial part and roots of *Sorghum Moench* (S) and *Panicum virgatum* (P) (g kg^{-1} d.m. of soil) in soil uncontaminated and contaminated with B, Zn^{2+} and B+Zn^{2+}

Objects	*Sorghum Moench* (S)		*Panicum virgatum* (P)	
	aerial parts of plants	roots	aerial parts of plants	roots
C	15.523^{b}	6.163^{b}	13.107^{bc}	4.180^{c}
B	12.260^{b}	2.140^{cd}	7.167^{cd}	1.817^{d}
Zn	1.663^{c}	1.137^{de}	2.330^{c}	0.470^{e}
B_Zn	1.160^{c}	0.143^{e}	2.623^{c}	0.603^{e}
C_H	23.047^{a}	10.067^{a}	31.523^{a}	11.570^{a}
B_H	11.740^{b}	3.267^{c}	19.883^{b}	6.530^{b}
Zn_H	1.157^{c}	0.087^{e}	5.380^{cd}	1.680^{de}
B_Zn_H	1.740^{c}	0.077^{e}	4.873^{cd}	1.247^{d}

C- uncontaminated soil, H – humic acid, S - *Sorghum Moench*, P - *Panicum virgatum,* B – bisphenol A, Zn – ion zinc (Zn^{2+}). Homogeneous groups denoted with letters (a-e) were calculated separately for each part of plant and plants species.

Table S5. The mean values of SPAD on the 50th day of growth of *Sorghum Moench* (S) and *Panicum virgatum* (P) in the soil contaminated with B, Zn^{2+} and B+Zn^{2+}

Objects	*Sorghum Moench* (S) BBCH 50	*Panicum virgatum* (P) BBCH 50
C	20.55^{c}	18.35^{e}
B	32.71^{b}	26.52^{d}
Zn	31.23^{b}	29.85^{d}
B_Zn	31,20^{b}	17.01^{e}
	Sorghum Moench (S)+H BBCH 50	*Panicum virgatum* (P)+H BBCH 50
C	48.72^{a}	34.99^{c}
B	44.90^{a}	43.38^{b}
Zn	28.33^{bc}	49.65^{a}
B_Zn	30.00^{bc}	44.19^{b}

C – uncontaminated soil, S -*Sorghum Moench*, P -*Panicum virgatum*, H – humic acid, B – bisphenol A, Zn – ion zinc (Zn^{2+}). Homogeneous groups denoted with letters (a-d) were calculated separately for each plant species.

Table S6. Physicochemical properties, enzyme activity and the number of microorganisms in the soil prior to setting up the experiment

Parameters	Unit	Values
pH_{KCl}	mol KCl dm^{-3}	4.4
HAC		26.1
EBC	mmol (+) kg^{-1} d.m. of soil	63.6
CEC		89.7
BS	%	70.90
C_{org}	g kg^{-1} d.m. of soil	10.00
N_{total}		0.83
$Na_{aveilable}$		10.00
$K_{aveilable}$	mg kg^{-1} d.m. of soil	168.00
$Mg_{aveilable}$		82.10
enzymes activity, kg^{-1} d.m. of soil h^{-1}		
Deh	μmol TFF	3.088
Ure	mmol N-NH_4	0.478
Cat	mol O_2	0.354
Pal		0.534
Pac	mmol 4-nitrofenol (PN)	3.409
Aryl		0.434
Glu		0.381
The number of microorganisms, kg^{-1} d.m. of soil		
Org		41.030
Act		108.809
Fun	cfu 10^{n}	22.124
Ps		44.985
Art		67.847
Cel		54.572

pH_{KCl} - soil reaction, HAC - hydrolytic acidity, EBC – sum of exchangeable base cations, CEC - cation exchange capacity, BS - base saturation, C_{org}—total organic carbon; N_{total}—total nitrogen; Deh - dehydrogenases; Ure - urease, Pal – alkaline phosphatase, Pac – acid phosphatase, Aryl - arylsulfatase, Glu – *β*-glucosidase; Org – organotrophic bacteria, Act - actinomycetes, Fun - fungi, Ps - *Pseudomonas* sp., Art - *Arthrobacter* sp., Cel – cellulolytic bacteria, n-exponent 7 for Org, Act, Cel, 6 for Fun, Ps, Art.

Table S7. Methodology for determining the activity of soil enzymes

Enzyme	Substrate	Product/unit	Wavelength λ (nm)	References
Deh (EC 1.1)	2,3,5-Triphenyl tetrazolium chloride (TTC)	Triphenyl fomazan (TFF)/ μmol kg^{-1} DM of soil h^{-1}	485	[110]
Ure (EC 3.5.1.5)	Urea	N-NH_4/ mmol kg^{-1} DM of soil h^{-1}	410	[111]
Glu (EC 3.2.1.21)	4-Nitrophenyl-β-D-glucopyranoside (PNG)	4-Nitrophenol (PN)/ mmol kg^{-1} DM of soil h^{-1}	420	[111]
Pac (EC 3.1.3.2)	Disodium 4-nitrophenyl phosphate hexahydrate (PNP)	4-Nitrophenol (PN)/ mmol kg^{-1} DM of soil h^{-1}	410	[111]
Pal (EC 3.1.3.1)	Disodium 4-nitrophenyl phosphate hexahydrate (PNP)	4-Nitrophenol (PN)/ mmol kg^{-1} DM of soil h^{-1}	410	[111]
Aryl (EC 3.1.6.1)	Potassium-4-nitrophenylsulfate (PNS)	4-Nitrophenol (PN)/ mmol kg^{-1} DM of soil h^{-1}	400	[111]
Cat (EC 1.11.1.6)	H_2O_2—aqueous solution	O_2/ mol kg^{-1} DM of soil h^{-1}	-	[111]

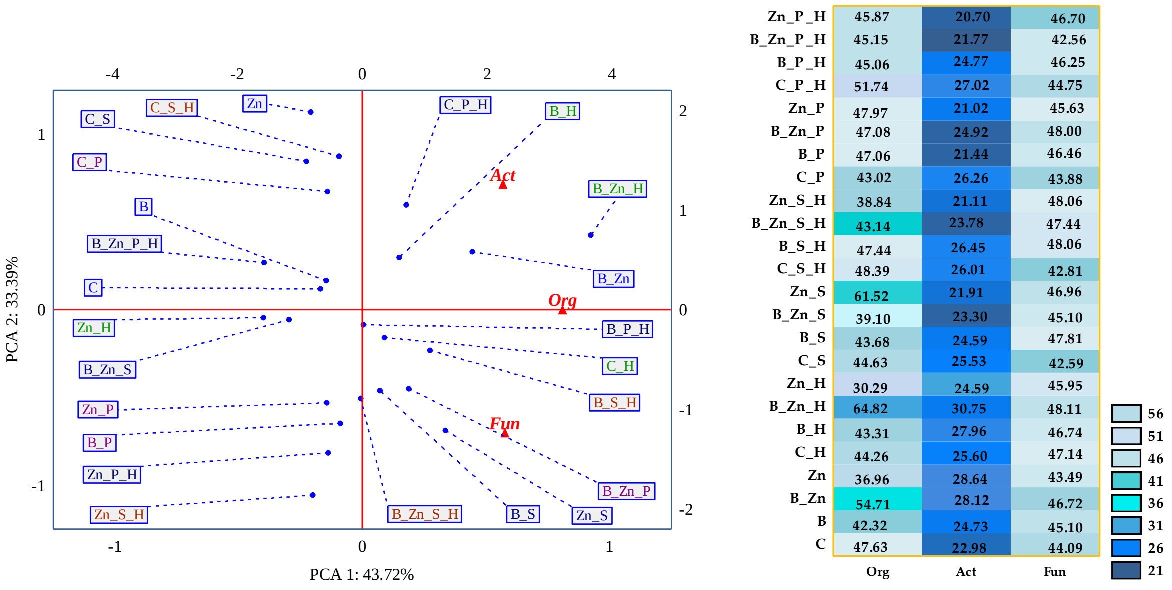

a) **b)**

Figure S1. Influence of soil contamination with B, Zn^{2+}, and B+Zn^{2+} on the colony development index (CD) of organotrophic bacteria, (Org) actinomycetes (Act) and fungi (Fun), dependencies between index values – PCA method (a), index value heatmap (b); C - uncontaminated soil, H – humic acid, S – *Sorghum Moench*, P – *Panicum virgatum*; B – bisphenol A, Zn – ion zinc (Zn^{2+}).

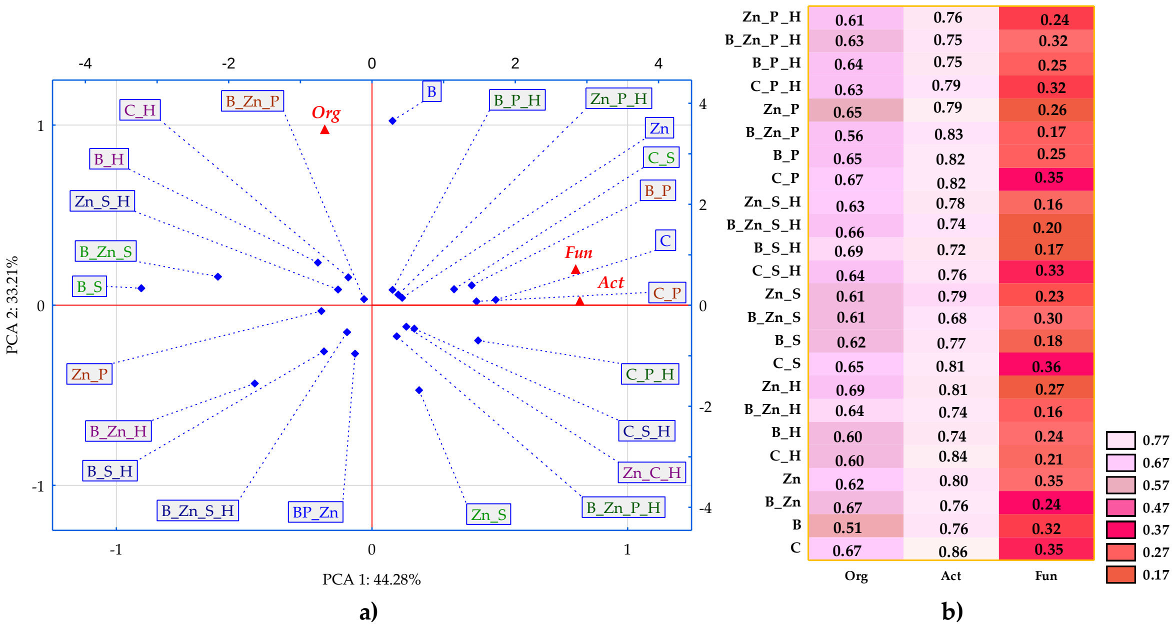

Figure S2. Influence of soil contamination with B, Zn^{2+}, and B+Zn^{2+} on the ecophysiological diversity index (EP) of organotrophic bacteria, (Org) actinomycetes (Act) and fungi (Fun), dependencies between index values – PCA method (a), index value heatmap (b),_C- uncontaminated soil, H – humic acid, S - *Sorghum Moench*, P - *Panicum virgatum*, B – bisphenol A, Zn – ion zinc (Zn^{2+})
